# Quantification of Photoreceptors’ Changes in a Diabetic Retinopathy Model with Two-Photon Imaging Microscopy

**DOI:** 10.3390/ijms25168756

**Published:** 2024-08-11

**Authors:** Nazario Bautista-Elivar, Marcelino Avilés-Trigueros, Juan M. Bueno

**Affiliations:** 1 Departamento de Ingeniería Eléctrica y Electrónica, Tecnológico Nacional de México/Instituto Tecnológico de Pachuca, Pachuca 42082, Hidalgo, Mexico; 2 Departamento de Oftalmología, Facultad de Medicina, Universidad de Murcia e Instituto Murciano de Investigación Biosanitaria Virgen de la Arrixaca, “Campus Mare Nostrum” de Excelencia International, 30100 Murcia, Spain; 3 Laboratorio de Óptica, Instituto Universitario de Investigación en Óptica y Nanofísica, Universidad de Murcia, 30100 Murcia, Spain

**Keywords:** two-photon microscopy, diabetic retinopathy, neuroinflammation, photoreceptors

## Abstract

Emerging evidence suggests that retinal neurodegeneration is an early event in the pathogenesis of diabetic retinopathy (DR), preceding the development of microvascular abnormalities. Here, we assessed the impact of neuroinflammation on the retina of diabetic-induced rats. For this aim we have used a two-photon microscope to image the photoreceptors (PRs) at different eccentricities in unstained retinas obtained from both control (N = 4) and pathological rats (N = 4). This technique provides high-resolution images where individual PRs can be identified. Within each image, every PR was located, and its transversal area was measured and used as an objective parameter of neuroinflammation. In control samples, the size of the PRs hardly changed with retinal eccentricity. On the opposite end, diabetic retinas presented larger PR transversal sections. The ratio of PRs suffering from neuroinflammation was not uniform across the retina. Moreover, the maximum anatomical resolving power (in cycles/deg) was also calculated. This presents a double-slope pattern (from the central retina towards the periphery) in both types of specimens, although the values for diabetic retinas were significantly lower across all retinal locations. The results show that chronic retinal inflammation due to diabetes leads to an increase in PR transversal size. These changes are not uniform and depend on the retinal location. Two-photon microscopy is a useful tool to accurately characterize and quantify PR inflammatory processes and retinal alterations.

## 1. Introduction

Diabetic retinopathy (DR) is a common complication of diabetes and the leading cause of vision loss in working-age individuals in many industrialized countries [1]. Although DR is clinically defined as a microvascular disease [2,3,4], recent studies have reported the presence of neuroretinal changes [5,6]. Different works suggest that DR produces sophisticated neurovascular complications including the disruption of interactions among multiple types of cells (neurons, glia and vascular cells) [7]. This deterioration of the neural retina may occur before microvascular changes [8].

Inflammation is a protective response of the immune system to harmful stimuli, and its principal function is to localize and minimize the damage to restore tissue homeostasis [9]. In the retina, microglia have been recognized as a pivotal factor in maintaining eye homeostasis [10]. These microglia are the key players in retinal inflammation since they activate to exert neuroprotection for degenerating photoreceptor (PR) cells [11]. Retinal neuroinflammation is a common pathogenic marker of various chronic ocular diseases, such as DR and retinitis pigmentosa, and this is strongly associated with cell death, especially in a necrotic form [12,13].

Local inflammation of the retinal tissue is a major risk factor in the development and progression of DR [14]. Among the different retinal cells, PRs are a significant source of diabetes-induced oxidative stress and local inflammation [15]. As a result, DR is considered a chronic inflammatory disorder [14,16,17]. It has been observed that individuals suffering from DR experience neurosensory deficits due to inflammation, even before any vascular complication becomes clinically identifiable [18]. Then, as the disease progresses, the retina presents characteristics related to both chronic states of neuroinflammation and vasculopathy [16]. Although the exact link between DR inflammatory disturbances and neural function loss is still unclear, there is mounting evidence that inflammatory processes play a significant role in the sequence of pathological changes produced during the development of the retinal disease [19,20].

Streptozotocin (STZ) is an antimicrobial agent used for inducing diabetes in animal models [21]. Initial damage occurs within 48 h of drug inoculation. Throughout this period, increased blood glucose levels and changes in insulin status are developed [21,22]. The most immediate effect is the selective destruction of pancreatic ß cells, which causes insulin deficiency [22,23]. STZ-induced diabetes is also characterized by enlarged oxidative stress [22,24] and the appearance of various biomarkers of inflammation in the retina [25]. PR degeneration has also been identified as a complication during the progression of DR [26].

Abcouwer and Gardner considered that diabetes causes measurable dysfunctions in the complex integral network of retinal cells and neurovascular structures [27]. In particular, they evidenced inflammation in the neuritis (axons and dendrites) and cell bodies of retinal neurons. Since this diabetic-related retinal neurodegeneration, neuroinflammation and deterioration of cell–cell interactions lead to visual disorders, it is crucial to use powerful imaging strategies to accurately visualize retinal cells and evaluate their spatial distribution under those pathological conditions. This will help to further understand the origin, effects and development of DR.

In this sense, this work analyzes the neuroinflammatory effects produced in the retinal PRs during the development of STZ-induced DR in a rat model. The impact of inflammation on the retina was assessed by determining the transversal size of the PRs. To the best of our knowledge, this is the first time that this parameter has been used to objectively measure DR neuroinflammation effects.

Images of the PRs were acquired using two-photon excitation fluorescence (TPEF) microscopy. This is a nonlinear imaging modality based on the quasi-simultaneous absorption of two infrared photons by the tissue under analysis to emit only one visible photon [28]. Since these photons are generated by endogenous fluorophores (proteins, mitochondrial NAD(P)H, flavins, …), the use of markers is not required. Furthermore, the TPEF signal is limited to the focal spot of the microscope objective, which provides inherent confocality, sectioning capabilities, enhanced depth penetration and reduced photodamage [28]. For almost 20 years, TPEF microscopy has provided high-resolution single-cell-resolved images of the different retinal layers in non-stained samples from both humans and animal models [29,30,31,32]. Although in vivo applications have already been reported, their use in clinical environments is not available at the moment [33,34].

## 2. Results

**Figure 1:**
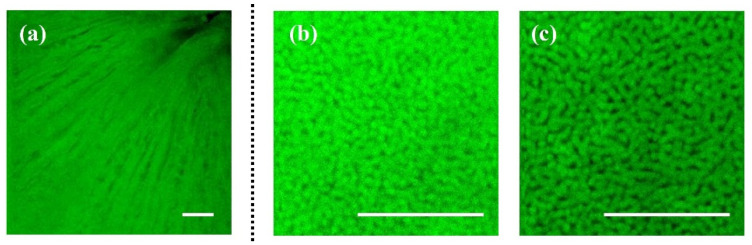
Examples of TPEF microscopy images of the retinal nerve fiber layer in a control sample (**a**), and the PR mosaic for a control (**b**) and a diabetic (**c**) rat retina. Images of the PRs shown herein were acquired at the same retinal location. Bar length: 50 μm.

Figure 1 presents illustrative examples of TPEF images acquired with our experimental system. These correspond to the retinal nerve fiber layer (Figure 1a) and to the PR layer in a control (Figure 1b) and a diabetic retina (Figure 1c). PR images correspond to the same retinal eccentricity and localization. Although individual PRs are clearly visualized, it is hard to detect differences between both PR mosaics by direct inspection. Differences can be inferred from the values of the cell density calculated as described in [32]: 43,951 and 33,210 cells/mm^2^, respectively, in this example.

**Figure 2:**
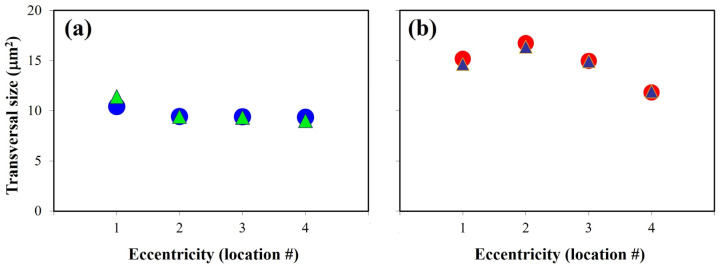
The PR transversal area (in μm^2^) as a function of retinal eccentricity for two different retinal quadrants (SN, circles and IT, triangles) in a control (**a**) and a diabetic retina (**b**). The maximum differences across the four locations were 2.4 and 4.5 μm^2^ in (**a**) and (**b**), respectively.

Figure 2 depicts the transversal area (in μm^2^) of the PRs in a control (Figure 2a) and a diabetic retina (Figure 2b) along the four retinal locations for two different quadrants. For both retinas, the two sets of data showed no significant differences (*t*-test, *p* = 0.399 and 0.443, respectively).

For the control retina, there are hardly any differences in the PR transversal area across locations. The values ranged between 9.0 and 11.4 μm^2^ for this particular specimen. On the contrary, in the diabetic retina, the area values presented a larger range of variability, with a minimum of ~12 μm^2^ and a maximum of ~16.5 μm^2^. Those behaviors were similar for all specimens in the rest of the retinal quadrants.

**Figure 3:**
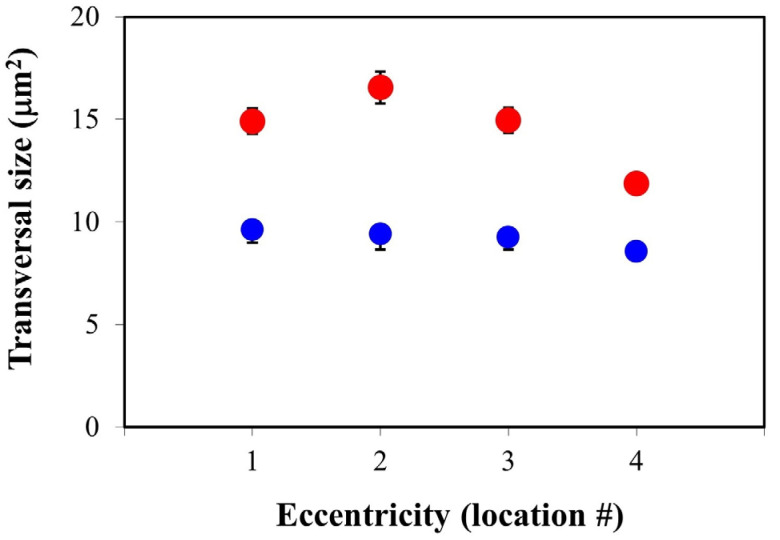
A comparison of the PR transversal areas as a function of the retinal eccentricity for diabetic (red symbols, N = 4) and control (blue symbols, N = 4) retinas. For every location, each symbol represents the mean across all specimens within the corresponding experimental group. The values for diabetic retinas were always larger and depended on the retinal location. The PR transversal size was similar for the control retinas..

Figure 3 shows the PR transversal size averaged across all specimens as a function of the retinal eccentricity in both control (blue) and diabetic eyes (red). As stated above, this transversal area was used as an objective parameter to quantify the effects of neuroinflammation. A visual inspection reveals that, independently of the retinal location, the values for diabetic retinas are well above those of the control samples, which might be associated with the inflammation produced in the retinal cells because of diabetes. The mean size for all retinas and locations was 10.04 ± 0.42 and 14.82 ± 1.49 μm^2^ for the control and pathological retinas, respectively. 

At every eccentricity, differences between both data sets achieved statistical significance (Mann–Whitney U test; *p* < 0.0001). In addition, for the control retinas, there were no differences among locations. On the opposite end, in the pathological retinas, the size increases from location #1 to #2 and decreases when moving towards locations #3 and #4.

**Figure 4:**
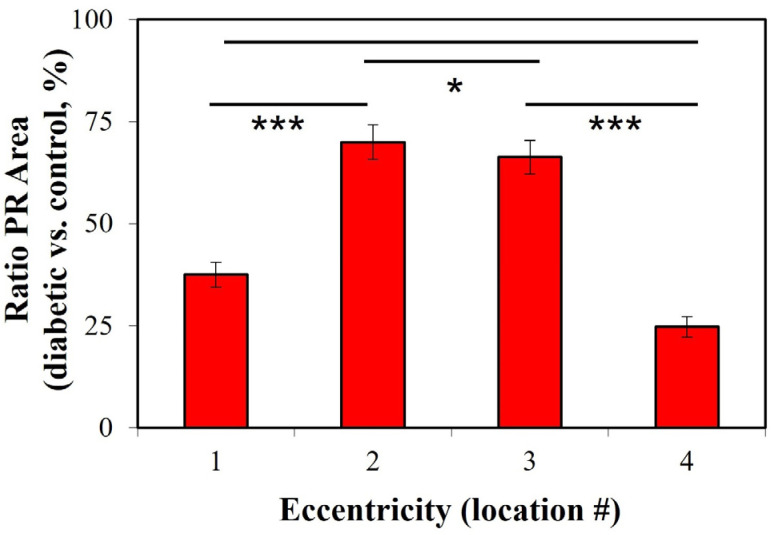
The ratio of transversal PR size (diabetic vs. control, in %) for every retinal eccentricity. Each bar corresponds to the value for a particular retinal location averaged across all retinas. (***: *p* < 0.0001; *: *p* = 0.023).

For a better description and understanding of the effects produced by diabetes on the enlargement of the PR transversal area, the ratio of diabetic vs. control (in %) is depicted in Figure 4. As expected from the previous figures, there is some variability among the different locations, and the values ranged between a minimum of 25% for location #4 and a maximum of 70% for #2. The differences between locations #2–#3 and #1–#4 were statistically different (*t*-test, *p* = 0.023). 

**Figure 5:**
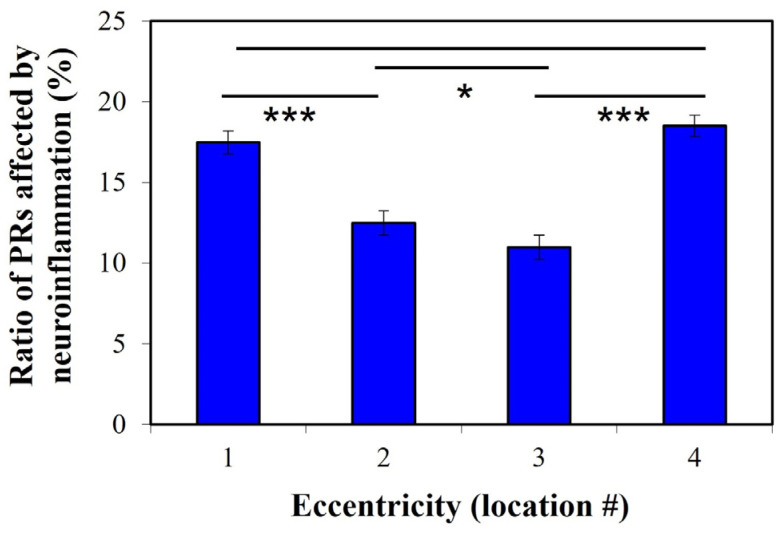
Percentage of PRs affected by neuroinflammation in diabetic retinas. For each individual TPEF image, the value was computed as the ratio (in %) between the number of PRs with neuroinflammation (i.e., those with a transversal area larger than 10 μm^2^) and the total number of PRs. Similar to the previous figure, the bar for each location represents the value averaged across all retinas (***: *p* < 0.0001; *: *p* = 0.029).

For each diabetic retina and eccentricity (i.e., from location #1 to #4), we identified and counted the PRs suffering from neuroinflammation within the corresponding TPEF image. These cells are those in which the transversal area was larger than the average value obtained for PRs in control retinas (10 μm^2^, i.e., “healthy threshold”). Then, the number of PRs with neuroinflammation over the total number of PRs within each TPEF image was computed. These results (in %) are those depicted in Figure 5. This plot shows that the fraction of PRs suffering neuroinflammation is higher at retinal eccentricities #1 and #4 (1.5 times) when compared to locations #2 and #3. The differences between both sets of locations were statistically significant (*t*-test, *p* = 0.029).

**Figure 6:**
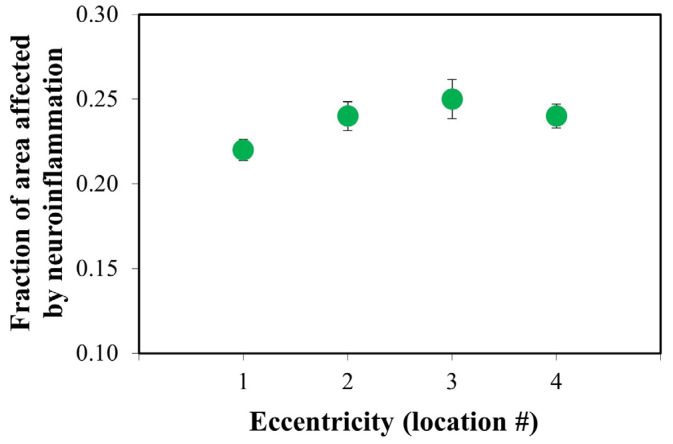
A fraction of the total area occupied by the PRs suffering from neuroinflammation for the different retinal locations. Each red symbol corresponds to the mean value for all diabetic retinas involved in this study. The parameter increases with the distance to the retina central area, reaching a maximum at location #3, then decreases towards location #4.

For completeness, for each location within every diabetic retina, the transversal area of all the PRs with a size larger than the “healthy threshold” was added up. This value was divided by the total area occupied by all the PRs across the entire TPEF image. This parameter provides information on the portion of the total area occupied by PRs affected by neuroinflammation. Figure 6 shows the results. The value increased from location #1 (i.e., near the optic nerve head) to location #3 where a maximum appears. Then, it decreased towards location #4 where the value is similar to that found at location #2.

**Figure 7:**
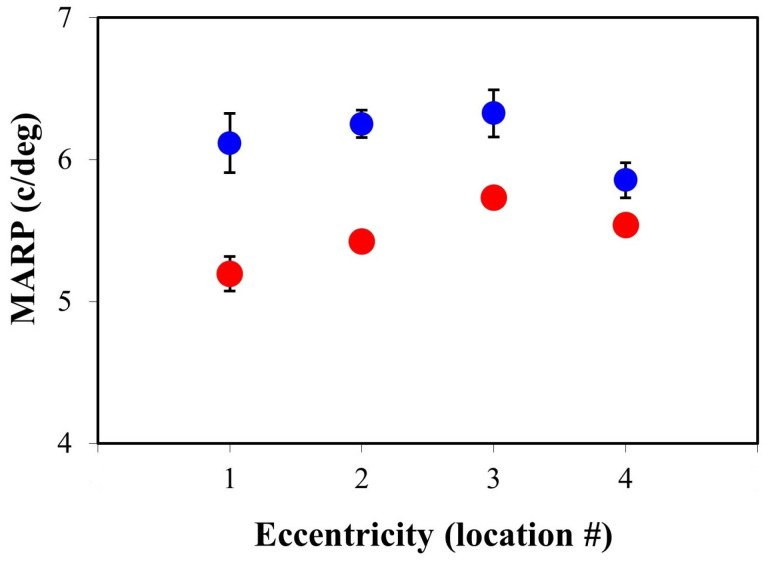
Values of the MARP (c/deg) in the rat retina as a function of retinal eccentricity computed using Equation (1), for both control (blue symbols) and diabetic (red symbols) eyes. Each symbol corresponds to the mean across all specimens for a particular retinal location within the corresponding group. The values for diabetic retinas were always smaller. The behavior for both experimental conditions was a double-slope pattern with a maximum appearing at location #3.

For each TPEF image, the MARP was computed as explained in Methods. Figure 7 shows the values as a function of the retinal location. As expected from the spatial distribution of PRs [32], the parameter does not decrease linearly towards the periphery. For both experimental conditions, MARP increases from location #1 to location #3 (a steeper slope in diabetic retinas) and then a fall appears at location #4. Moreover, for all retinal locations, the MARP was larger in the control eyes (12% on average), and this difference was statistically significant (*t*-test, *p* = 0.005).

## 3. Discussion

The mechanisms of retinal neurodegeneration are complex, likely multifactorial and might lead to retinal disorders [1,19]. Retinal degenerative and neovascular diseases, such as DR are the main causes responsible for global severe vision loss or blindness. Since DR has been reported to be more than just a neurovascular disorder [1,4,5], it is of great interest to develop new procedures of analysis and diagnosis to understand and potentially prevent neurodegeneration conditions during DR. In recent years, neuroinflammatory mechanisms have been identified as major factors for triggering the onset and progression of DR [13]. Additionally, although DR pathogenesis is understood, the sequence of the pathological changes occurring during DR development still remains unsolved [20].

Studies conducted on rodents have revealed that during 1 to 6 months of diabetes crisis, several biomarkers of inflammation appear in the retina [25]. Different cells involved in the DR inflammatory response process were also identified. These include astrocytes, Müller cells and those from the inner nuclear layer [35]. The inflammatory reactions that occur locally in the retina are mediated by activated microglia which inhabit the plexiform layers [36]. Ganglion cell body swelling and axonal fragmentation have also been demonstrated in a diabetic mouse model [27].

In a diabetic rat model (using an alloxan solution), transmission electron microscopy has shown that the pathology causes edema, which leads to a partial deformation and destruction of PRs [37]. With the same technique, Énzsöly et al. investigated neurodegeneration as an early event in STZ-induced DR rats [38]. They reported different degenerative changes in the PRs before apoptotic loss. This included inflammation of the inner segments and variations in the outer segment morphology, among others. More recently, the impact of inflammation on the retina was also assessed in mice by determining the length of the PR outer segments, the thickness of the outer and inner nuclear layer and the PR density [39]. Most of these previous studies have used wide-field, confocal or electron microscopes for the analysis of stained (i.e., immunohistochemistry) histological sections and flat-mounted specimens.

In this work, TPEF microscopy has been used to investigate neuroinflammatory processes in the retina of a diabetic rat model by measuring the transversal size of individual PRs. This is a powerful tool to image thick biological samples [28]. During the last few years, there has been an increasing interest in using this type of microscopy in analyses of ex vivo non-stained retinal tissues of different animal experiment models [29,30,31]. This technique provides high-resolution images where individual retinal cells are visualized and additional information on the spatial distribution can be obtained. The accurate visualization of retinal cells allowed for objective analyses and the calculation of different parameters, such as cell inter-distance, density, area and geometrical distribution, among others. 

In a rat model, significant differences in PR density and spatial arrangement between control and diabetic retinas have been reported in the past [32]. However, studies exploring the spatially resolved retinal structure in rats using TPEF microscopy are scarce in the literature. As far as these authors know, this is the first time that TPEF images have been used to quantify the changes in transversal size suffered by the PRs of an STZ-induced diabetic rat model.

The results within the present work reveal that the transversal size of PRs in control specimens is fairly uniform across the retina (~10 μm^2^). However, for diabetic specimens, this size was significantly higher (48% on average) for all retinal eccentricities. Furthermore, no statistical differences were found among the four retinal quadrants in either the control or diabetic retinas. Due to the advanced diabetic stage of the retinas employed herein, this increase in PR size might be associated with the neuroinflammation effects resulting from the pathology.

It is interesting to note that, unlike control retinas, the transversal area in diabetic tissues is less uniform across retinal eccentricities. From values around 15–16 μm^2^ at locations #1–3, the value decreases to ~12 μm^2^ at location #4. The differences between this peripheric location and the rest were statistically significant. It has also been shown that the ratio of PR affected by neuroinflammation is not uniform but depends on the retinal location, being significantly higher at both lower and larger eccentricities.

Although the presence, and overexpression, of inflammatory proteins such as iNOS and COX2 have been demonstrated in the PRs of diabetic mice, this expression is confined to the inner segment of the PRs [40]. In our case, the TPEF image corresponds to the level of the PR nuclear layer. Therefore, direct comparisons of our results with those obtained using immunohistochemical techniques that detect the presence of those inflammatory proteins in the inner segment are not feasible.

The loss of PRs derived from DR affects both visual acuity and contrast sensitivity [41,42,43]. Visual tests are important to analyze the different stages of diabetic eye disease, to identify profiles of visual function or to develop prediction models [44]. Visual function impairment can have a considerable impact on the patient’s quality of life, particularly in the areas of independence, mobility, leisure and self-care activities [43,45]. Although this visual impairment was recognized early in DR human patients [46], measurements in animal models are more difficult. In STZ-induced diabetic rats, reduced visual acuity and contrast sensitivity were reported [47,48]. Unlike those previous works where optokinetic tracking in living animals was used, anatomically based values of visual resolution were computed herein. The parameter presents a double-slope pattern, and it does not decrease from the central to the peripheral retina (see Figure 7) as reported in humans or other animal models [31,49]. Moreover, visual resolution herein was significantly reduced in DR retinas when compared to control ones (12% on average).

In conclusion, TPEF microscopy has been used to objectively explore cellular inflammatory effects in diabetic rat retinas. High-resolution imaging allows us to accurately calculate the transversal area of individual cells and reveals inflammatory abnormalities across the neural retina. Diabetic retinas showed larger PRs for every retinal eccentricity and these inflammation effects were not uniform across the retina, which indicates that some retinal areas are more affected than others by the pathology. The results herein represent a step forward to understand the complex ocular inflammatory mechanisms. This type of microscopy might help to further quantitatively characterize the neurodegeneration changes suffered by the retina because of diabetes.

## 4. Material and Methods

### 4.1. Animal Model and Tissue Preparation

Eight male adult Wistar rats were used for the purpose of this work (weight ~200 g). Diabetes was induced in half of them by applying a unique dose of STZ diluted in citrate buffer (75 mg/kg), administered via intraperitoneal injection [50]. The rest of the animals (N = 4) were used as a control. All animals were housed in cages individually, under free access to food/water, and a 12 h light/dark cycle. After six weeks, 1 mL of blood was taken from the rat’s tail to measure glucose concentration in both non-injected and injected animals. Whereas normal levels of glucose were found in the control animals (<100 mg/dL), for the STZ-injected rats, the levels were noticeably higher (>200 mg/dL). Once chemical diabetes was confirmed, all animals were sacrificed, and the ocular globes were excised. One eye per animal was used herein (N = 4). Each retina was detached from the eye fundus, prepared and flat whole-mounted according to a well-established procedure [51]. Histological markers were not used. The entire experiment followed the guidelines of the Association for Research in Vision and Ophthalmology Statement for the Use of Animals in Ophthalmic and Vision Research. This study was approved by the Ethics Committee for Animal Research of the University of Murcia.

### 4.2. Experimental System

**Figure 8:**
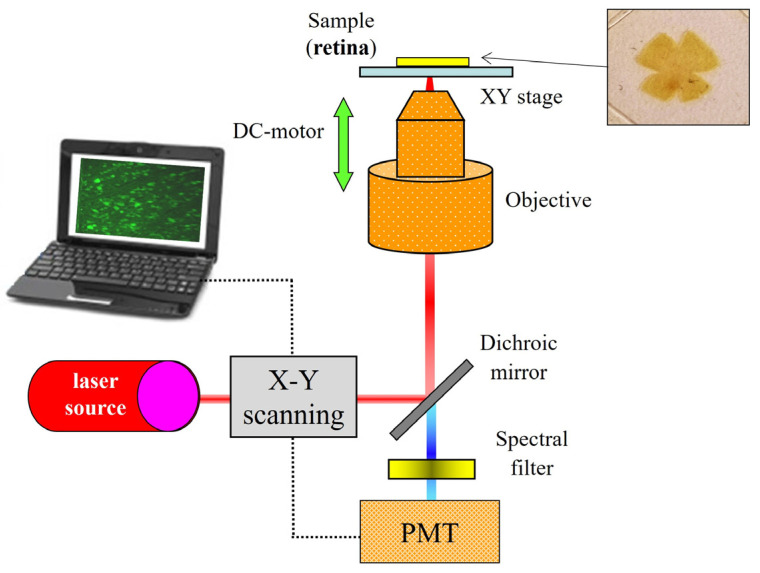
Optical setup of the two-photon imaging microscope used in this work. Along the incoming pathway, the laser beam passes an XY scanning unit, a dichroic mirror (to separate the ingoing infrared light from the outgoing visible light) and the objective. In the detection pathway, the emitted signal coming from the sample goes through the same objective and dichroic mirror, before passing a spectral filter and finally reaching the PMT.

The instrument is a research TPEF imaging microscope that combines a commercial inverted microscope (TE2000-U; Nikon Corp., Tokyo, Japan), an 800 nm Ti/sapphire laser, an XY scanning unit and a DC-motor (Figure 8). The illumination source is a femtosecond laser providing light pulses at a frequency of 76 MHz. This type of mode-locked laser is required to produce TPEF signals [28]. The beam passes the scanning unit and is focused onto the specimen via a dry long working-distance microscope objective (20x, NA 0.5). A sample’s area of 90 × 90 μm^2^ is scanned point by point. The signal emitted by each point is collected through the same objective (i.e., epi-collection) and filtered by an appropriate long-pass spectral filter before reaching the photon-counting photomultiplier tube (PMT) used as the detection unit. The entire instrument was controlled through home-made LabView^TM^ software (version 2010, Emerson; Austin, TX, USA). See [32,52] for further details on the microscope operation.

### 4.3. Image Acquisition, Processing and Analysis

Most retinal layers covering from the innermost retina (nerve fibers) to the PR outer segments can be visualized through TPEF microscopy [31,32,52]. However, our interest in the present work is centered on measuring the transversal area and spatial distribution of the PRs in both the control and diabetic rat retinas. This parameter is used as a measure of the neuroinflammatory effects occurring in the retina due to diabetes. Since samples used herein were unstained, the TPEF signals correspond to local endogenous fluorescence (i.e., autofluorescence) [53,54].

**Figure 9:**
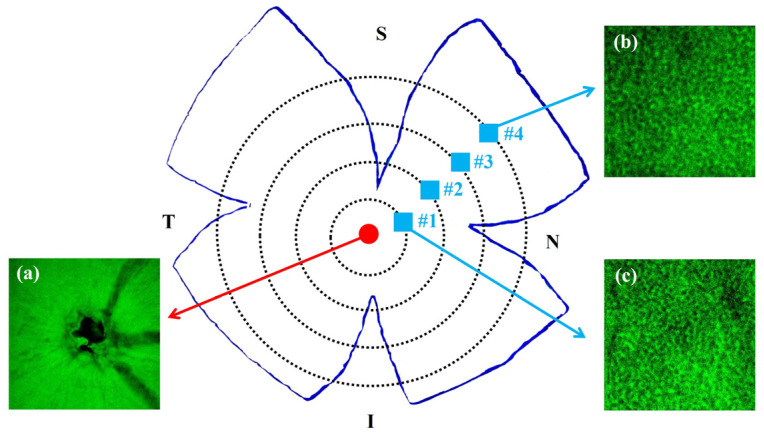
A schematic diagram of the retinal locations imaged in this study (named #1–#4). For simplicity, only the areas along the SN quadrant have been drawn. As an example, three representative TPEF images are also included: the optic nerve head (**a**) and the PRs from locations #1 (**b**) and #4 (**c**).

The four radial cuts made in the retinas using flat whole-mount preparation (see inset in Figure 8 and Figure 9) determined four oriented quadrants. These were named superior temporal (ST), superior nasal (SN), inferior temporal (IT) and inferior nasal (IN). For each quadrant, individual TPEF images of the PR mosaic at four different retinal eccentricities were acquired (numbered from #1 to #4). These locations were equi-spaced about 540 µm from the paracentral region (i.e., the area surrounding the rat’s optic nerve head) to the retinal periphery. For a better understanding, Figure 9 presents the distribution of the imaged areas across the retina together with some representative TPEF images.

**Figure 10:**
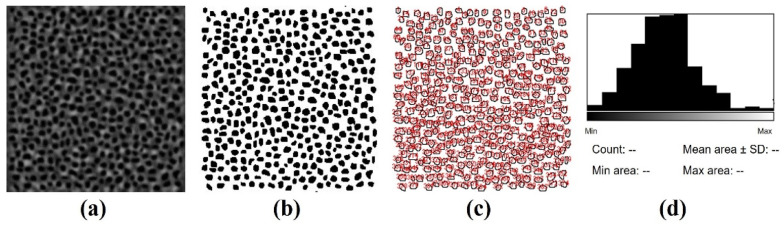
An example of the experimental procedure used to compute the PR transversal area. (**a**) A TPEF image with the corresponding manually tracked PRs; (**b**) the image obtained after contrast threshold and binarization; (**c**) watershed segmentation of the transversal PR area; and (**d**) a final histogram for PR counting and area computation.

For each image, the location of the individual PRs was manually tracked by the operator as previously described [31,32]. As an accuracy test, PR tracking was performed by two independent observers (co-authors) in 4 randomly chosen TPEF images (two from each group of specimens). Differences in the amount of PR never exceeded 5%. TPEF intensity provides a strong signal from the PR layer, and the individual cells were visible; however, to facilitate the method, an image contrast threshold and a binarization process were applied. Once the PRs were located, the public domain image processing software ImageJ (version ImageJ 1.53) was used to automatically segment individual PRs and compute each transversal area (in μm^2^). Figure 10 shows an example of the procedure in one of the acquired images.

Moreover, the maximum potential anatomical resolving power (MARP, in cycles/deg) of the rat eye was also computed for each TPEF image of the PR mosaic as follows [55]:

MARP = PND/(57.3⋅D_PR_⋅√3) (1)

where PND = 3.405 mm is the posterior nodal distance of the schematic rat eye model [56], and D_PR_ is the local average inter-center PR distance computed from the Fourier power spectrum of the images, as previously reported by Yellott [57].

The SPSS software (SPSS Statistics 28.0 software, IBM Corp.; Armonk, NY, USA) was used to perform the statistical analysis. Differences were considered significant when *p* < 0.05. The Kolmogorov–Smirnov test was used to check whether data distributions were normal. If so, the paired Student’s *t*-test was performed; otherwise, the Mann–Whitney U test was employed.

## Data Availability

Data underlying the results presented in this paper are not publicly available at this time but may be obtained from the authors upon reasonable request.
